# Interstitial Lung Disease and Profound Hypoxaemia in a Severely-malnourished Child with Very Severe Pneumonia and Potential Lymph-node Tuberculosis: An Uncommon but Serious Co-morbidity

**DOI:** 10.3329/jhpn.v31i1.14758

**Published:** 2013-03

**Authors:** Mohammod J. Chisti, Irin Parvin, Hasan Ashraf, Haimanti Saha, Fariha B. Matin, Mark A.C. Pietroni

**Affiliations:** icddr,b, GPO Box 128, Dhaka 1000, Bangladesh

**Keywords:** Hypoxaemia, Infant, Interstitial lung disease, Lymph-node tuberculosis, Severe malnutrition, Very severe pneumonia

## Abstract

A nine-month old boy was initially admitted at the Acute Respiratory Infection Unit of Dhaka Hospital of icddr,b and soon after transferred to the Intensive Care Unit of the same hospital. The boy had problems of very severe pneumonia (confirmed by radiology), severe hypoxaemia, severe malnutrition, and Down's syndrome. The patient was treated according to the hospital protocol for the management of pneumonia and malnutrition. During the hospital stay, hypoxaemia was persistent with very little improvement of pneumonia; a number of differentials, such as pneumocystis jirovecii pneumonia, lymph-node tuberculosis, were added to the problems. Subsequently, the patient's hypoxaemia improved with the empirical use of antitubercular drugs. However, the patient again developed persistent hypoxaemia and, after unsuccessful treatment for a hospital-acquired pneumonia, the problems further expanded to include interstitial lung disease (ILD). This was confirmed by high-resolution computed tomography, and the patient was treated with prednisolone for 6 months, along with antitubercular drugs. He fully recovered from ILD, hypoxaemia, and pneumonia both clinically and radiologically. Therefore, severely-malnourished children having wet cough and pneumonia with persistent hypoxaemia should be assessed for the possible existence of interstitial lung disease. This may help provide a prompt and appropriate management to reduce morbidity and deaths in such patients.

## INTRODUCTION

The clinical features of childhood tuberculosis (TB) often overlap with the features of pneumonia caused by other bacterial organisms, especially in children with severe malnutrition, complicating the diagnosis of TB in children based on clinical findings alone. Consequently, a large proportion of children with pulmonary or lymph-node TB may remain misdiagnosed as bacterial pneumonia, resulting in delays in initiating appropriate antituberculous treatment and a potentially fatal outcome. Lymph-node tuberculosis is the commonest form of extra-pulmonary TB in children living in TB-endemic areas and accounts for 8-10% of childhood TB cases in India and South Africa (1,2). The cervical lymph nodes are the commonest site of TB lymphadenitis whereas mediastinal lymphadenitis is comparatively less common (3). The diagnosis of active TB in adults is based on the isolation and culture of *Mycobacterium tuberculosis* from sputum and lymph nodes. However, in children with positive mycobacterial cultures are uncommon, making this not feasible as a diagnostic option. As a result, the diagnosis of childhood TB is often based on clinical features as well as reactive Tuberculin skin tests (TST), abnormal chest x-ray findings, and a history of contact with a known case of tuberculosis (4). In the TB-endemic areas, newly-formulated WHO scoring criteria and/or modified Kenneth Jones scoring criteria are often used for the clinical diagnosis of childhood TB. Children of less than 1 year of age have a risk of TB as high as 50%, and the risk is even higher for lymph-node TB (5). Children with very severe pneumonia and severe malnutrition with wet cough persisting for more than 3 weeks often have multiple co-morbidities, including childhood TB. We present the case of a 9-month old child having very severe pneumonia and severe malnutrition with lymph-node TB and interstitial lung disease (ILD).

## CASE HISTORY

A nine-month old boy from a poor family (monthly family income of Tk ~4,000), living in Valuka of Mymensingh district, was brought to the Dhaka Hospital of icddr,b in June 2010. He was admitted at the Longer Stay Acute Respiratory Infection (ARI) Unit of the Hospital, with a history of fever and wet cough for 6 days and breathing difficulty for the same duration. Fever was of low grade and intermittent in nature. He did not take any medicine at home. He was the only issue of his non-consanguineous parents and was delivered normally at home at full term. He was exclusively breastfed until nine months, and no complementary feeding was started at home. He was vaccinated as per EPI schedule. His birthweight and height could not be obtained. He had a history of pneumonia at five months of age. According to the mother's statement, he did not have a history of contact with any persons known or suspected to have tuberculosis. The parents were illiterate; the father was a farmer, and the mother was a housewife. The boy had passed urine just before admission at the ARI Unit.

On admission, the boy weighed 5.1 kg, with a length of 65 cm; his weight-for-age z-score was −5.00 and weight-for-length z-score was −4.72 of the median of the World Health Organization (WHO) growth standard. He was less active, and his rectal temperature was 38 °C. He had flat facies, upward-slanting eyes, a small nose with a flat nasal bridge, hypotonia, and simian creases. He had a regular normal-volume radial pulse of 170 per minute; his blood pressure was not recorded. He was not pale, cyanosed, or jaundiced, and there was no oedema, or clubbing. The boy's respiration rate was 72 per minute (normal respiration rate, as defined by the WHO, is <50 per minute for children aged 2 months to <12 months). There was marked use of the accessory muscles of respiration, and the patient had nasal flaring, head-nodding, and severe lower chest-wall in-drawing. His trachea was centrally placed, no abnormality was detected on chest percussion, and the respiratory sound was vesicular with bilateral rales and rhonchi which were more pronounced on the right side. Findings of other systemic examinations of the child were normal. His blood glucose, measured at the bedside, was 8.8 mmol/L, and his arterial oxygen saturation (SPO_2_) was 73% in room-air (WHO defines SPO_2_ of <90% at sea-level as hypoxaemia).

The initial problems were: (a) very severe pneumonia, (b) severe hypoxaemia, (c) severe protein-energy malnutrition, and (d) Down's syndrome.

**Figure 1.**
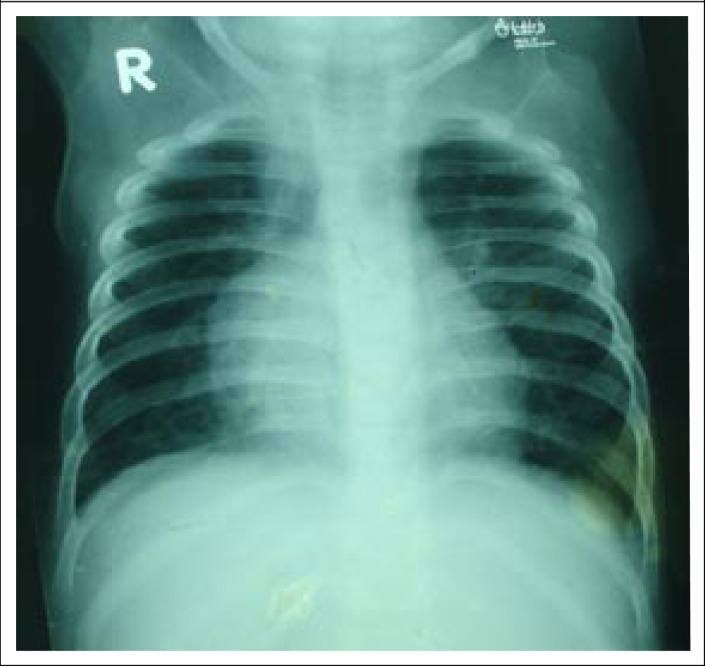
CXR on admission (bilateral pulmonary inflammatory lesion with enlarged mediastinal lymph nodes)

The laboratory tests included: blood for total and differential white blood cell (WBC) count, blood culture and sensitivity, serum electrolytes, creatinine, and a chest x-ray (Figure 1) to diagnose/exclude pneumonia. Broad-spectrum antimicrobial coverage was initiated using parenteral ceftriaxone and levofloxacin; magnesium sulphate, oral zinc, multivitamin, folic acid, potassium, and vitamin A were also given. His total WBC was 15,790/mm^3^, with 56% neutrophils, 36% lymphocytes, and 7% monocyte; serum sodium was 131.0 mmol/L, serum potassium 4.34 mmol/L, TCO_2_ 18.24 mmol/L; and serum creatinine was 33.7 micromole/L. The chest x-ray revealed consolidation (differentially, an enlarged mediastinal lymph node) in the right lung field. No organism was isolated in blood culture.

Nine hours after admission, the patient was transferred to the Intensive Care Unit (ICU) due to decreasing oxygen saturation despite getting oxygen via nasal prongs, and there was also evidence of heart failure (tachycardia, tachypnea, enlarged liver, and bilateral rales). At the ICU, his heart failure was managed with intravenous furosemide. Within the next 72 hours, the patient's general condition improved, although respiratory distress and hypoxaemia were persisting. However, the patient was maintaining a normal oxygen saturation with added oxygen (2L/min), and he was transferred to a high-dependency unit (HDU). During the next few days, his condition was static but on the 7^th^ day of admission, his condition became worse, the oxygen saturation dropped to around 65% without O_2_, although he maintained a normal saturation with O_2_; fever persisted, and there was also radiological deterioration of the pneumonia. The antibiotics were changed to Inj. Ceftazidime and Inj. Amikacin, and the investigations (blood culture, urine for routine microscopic examination and culture, serum electrolyte, and serum creatinine) were repeated. Thyroid function tests [serum level of thyroid-stimulating hormone (TSH), free tri-iodothyronine (FT3); free thyroxine (FT4)] and echocardiogram were also done to exclude hypothyroidism and congenital heart disease respectively (as these are associated with Down's syndrome) and were normal.

As there was persistent hypoxaemia with low-grade fever even after getting adequate antibiotic coverage, pneumocystis jirovecii pneumonia, or tuberculosis were considered and, on the 10^th^ day of admission, oral cotrimoxazole was added. By this time, all relevant laboratory investigations (Tuberculin skin test, Acid fast bacilli from gastric lavage) for tuberculosis were negative. His total score by the Modified Keneath Jones criteria, the clinical basis of anti-TB therapy in the hospital, was +4 (age below 2 years: +1, non-response to therapy: +1, repeat CXR showed persistent lymphadenopathy: +2, severe malnutrition: +1 and BCG given: −1) and two WHO criteria were positive (cough more than 3 weeks plus radiological evidence of patchy consolidation). To initiate anti-TB treatment, at least a score of 5 for Modified Keneath Jones criteria or 3 positive WHO criteria are required. However, empirical therapy with anti-TB drugs was initiated on the 14^th^ day of hospitalization due to the persistence of hypoxaemia despite cotrimoxazole.

After starting anti-TB drugs, the patient's clinical condition gradually improved, the hypoxaemia and respiratory distress resolved, and the lungs became clear on auscultation, although the wet cough persisted.

**Figure 2.**
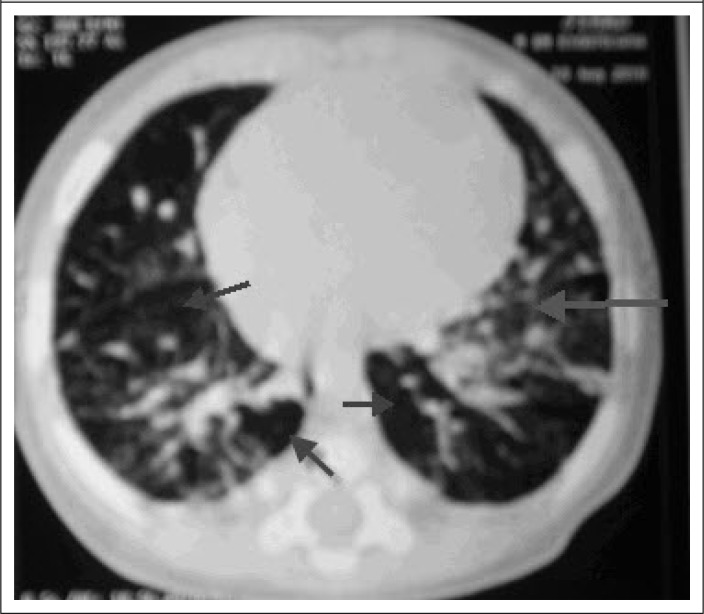
HRCT during the time of diagnosis [ground glass opacities (large arrow) with areas of low attenuation air spaces (air-trapping) (small arrows)]

On the 31^st^ day of admission, the boy's condition deteriorated again with respiratory distress, hypoxaemia and rales in the right lung on auscultation. The chest x-ray was repeated, which showed consolidation in the right apical zone, which was treated as a hospital-acquired pneumonia with Inj. Imipenam, in addition to ongoing antitubercular drugs. However, there was little improvement of the clinical signs after adding Inj. Imipenam, and the hypoxaemia was still persisting. Finally, high-resonance computer tomography (HRCT) was done (Figure 2) to exclude chronic suppurative or interstitial lung disease (ILD). The HRCT showed consolidation in the right-upper lobe with interstitial pneumonitis having the features of air-trapping―a variety of ILD. Tablet prednisolone (2 mg/kg/day once daily) was added on the 45^th^ day of hospitalization in addition to ongoing antitubercular drugs. The hypoxaemia gradually improved, and the patient was discharged after 53 days of admission, with a final diagnosis of lymph-node tuberculosis, along with very severe pneumonia, interstitial lung disease, severe malnutrition, and Down's syndrome.

**Figure 3.**
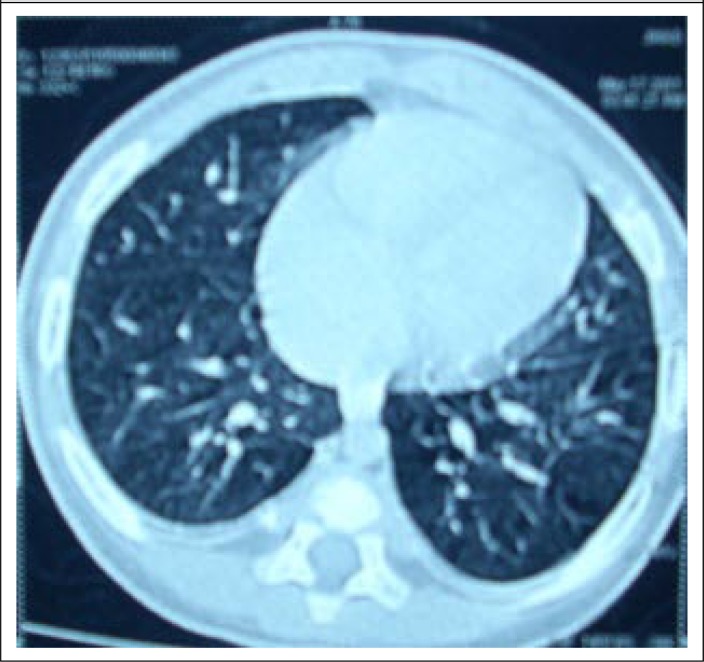
Follow-up HRCT (almost normal chest in HRCT, except mild bilateral pleural thickening)

The steroid were continued for three months and then tapered over the next 3 months. The HRCT was repeated (Figure 3) after 6 months when it was normal. The boy was doing well at home without any medication. He was advised to attend the Shishu Bikash Kendra of the Dhaka Shishu Hospital for long-term management of his Down's syndrome.

## DISCUSSION

An uncommon case of interstitial lung disease (ILD), along with severe persistent hypoxaemia and Down's syndrome in a severely-malnourished child who initially presented with very severe pneumonia and suspected lymph-node tuberculosis is reported here for the first time in medical literature. The aetiology of pneumonia in severely- malnourished children is quite different from that in children without severe malnutrition (6). TB is commonly found to be one of the aetiologic agents of acute pneumonia in severely-malnourished children (6), and a large proportion of these present in the form of TB lymphadenitis (3). TB lymphadenitis in the majority of cases represents the glandular component of a primary complex, which consists of the Ghon focus (or site of primary infection) usually in the regional lymph nodes (3,7). In our case, the regional lymph node of the Ghon focus was probably the mediastinal lymph nodes where there was a persistent visible opacity consistent with enlarged mediastinal lymph nodes in all of the chest radiographs performed during the hospital stay of the patient. Although TST was not suggestive of TB, persistent radiographic signs suggestive of lymphadenitis in TB-endemic countries support the diagnosis of TB lymphadenitis (8). In our case, severe malnutrition, mainly due to the lack of proper complementary feeding even at 9 months of age, probably resulted in the failure to achieve an adequate inflammatory response (9). Severe TB without a positive TST due to a poor inflammatory response is not uncommon in this type of severely-malnourished child―potentially a major problem for the appropriate and timely management of such children (9,10). Although TB in mediastinal lymph nodes is less common (3) and the Modified Keneath Jones (10) and WHO criteria (11) did not overwhelmingly support the diagnosis of TB lymphadenitis in our case, the clinical exclusion of all other relevant diagnoses (such as bacterial pneumonia and pneumocystis jerovicii pneumonia) encouraged us to initiate empirical antitubercular therapy to which the patient subsequently responded very well. Interestingly, hypoxaemia is a common feature in severely-malnourished children presenting with community-acquired very severe pneumonia (12) and hospital-acquired pneumonia (13) but subsequent persistent and profound hypoxaemia in mediastinal TB is not so common.

Congenital heart disease is quite common in Down's syndrome (14) but could not be detected by echocardiogram in our patient, thus, excluding this as a cause of the persistent hypoxaemia. However, the persistence of wet cough for more than a month in a child with Down's syndrome is usually associated with hypoplastic lung and rapidly-progressing pulmonary hypertension and repeated respiratory tract infections (15). This ultimately leads to interstitial lung disease (ILD) or chronic suppurative lung disease, such as protracted bronchitis, chronic suppurative bronchitis, bronchiectasis, or immunodeficiency (16). Hypoxaemia should be absent in protracted bronchitis; thus, the presence of severe and persistent hypoxaemia excluded probability of the existence of protracted bronchitis in our case (16); the history did not support a diagnosis of immunodeficiency, such as HIV. HRCT excluded chronic suppurative bronchitis and bronchiectasis (16) and simultaneously confirmed the diagnosis of interstitial pneumonitis consistent with ILD. Although very little is known about interstitial pneumonitis in children, it has been categorized as one of the acute forms of ILD. It is a rapidly-progressing form of parenchymal lung disease that results from diffuse alveolar damage and is potentially similar to the fibrotic phase of adult respiratory distress syndrome which usually takes about a month to develop (17) as in our case. Although there is no specific proven therapy for children, our trial of therapy with oral prednisolone (2 mg/kg/dose) (17) led to a dramatic improvement of the ILD confirmed by the resolution of findings on the repeated HRCT. The remission of the ILD with oral prednisolone therapy indicates that the ILD occurred probably due to a rapidly-progressing acute parenchymal infection (17). Simultaneously, prednisolone might have effectively reduced inflammation by reducing neutrophilia in the alveolus with a very good future prognosis (17). The Down's syndrome remains a great threat to his cognitive and other development. A multidisciplinary approach, including social engagement, feeding, physiotherapy, continued assurance to the parents, which are common components in the successful management of children with Down's syndrome in developed countries (14), is almost absent in our country. However, the patient has been referred to the Shishu Bikash Kendra, a child unit in a children's hospital in Dhaka which deals with handicapped children, including those with Down's syndrome aiming to improve their cognitive function as well as quality of life.

### Conclusions

The presence of co-morbidity—very severe pneumonia, severe malnutrition, interstitial lung disease, and potential lymph-node tuberculosis with profound hypoxaemia in a child having chronic wet cough and Down's syndrome—is the remarkable observation in this case study. Thus, physicians should consider ILD as well as TB in the differential to institute early and proper management to reduce morbidity and mortality in such children.
